# Antenatal Corticosteroids: Extending the Practice for Late-Preterm and Scheduled Early-Term Deliveries?

**DOI:** 10.3390/children8040272

**Published:** 2021-04-01

**Authors:** Zeyar T. Htun, Jacqueline C. Hairston, Cynthia Gyamfi-Bannerman, Jaime Marasch, Ana Paula Duarte Ribeiro

**Affiliations:** 1 Division of Neonatology, Rainbow Babies and Children’s Hospital, Cleveland, OH 44106, USA; jaime.marasch@uhhospitals.org (J.M.); ana.ribeiro@uhhospitals.org (A.P.D.R.); 2 Department of Obstetrics and Gynecology, Division of Maternal-Fetal Medicine, Columbia University Irving Medical Center, New York, NY 10032, USA; jh4298@cumc.columbia.edu (J.C.H.); cg2231@cumc.columbia.edu (C.G.-B.); 3 Department of Pharmacy, Rainbow Babies and Children’s Hospital, Cleveland, OH 44106, USA

**Keywords:** antenatal corticosteroids, late-preterm, early term, caesarean delivery, RDS

## Abstract

Respiratory distress in late-preterm and early term infants generally may warrant admission to a special care nursery or an intensive care unit. In particular, respiratory distress syndrome and transient tachypnea of the newborn are the two most common respiratory morbidities. Antenatal corticosteroids (ACS) facilitate surfactant production and lung fluid resorption. The use of ACS has been proven to be beneficial for preterm infants delivered at less than 34 weeks’ gestation. Literature suggests that the benefits of giving antenatal corticosteroids may extend to late-preterm and early term infants as well. This review discusses the short-term benefits of ACS administration in reducing respiratory morbidities, in addition to potential long term adverse effects. An update on the current practices of ACS use in pregnancies greater than 34 weeks’ gestation and considerations of possibly extending versus restricting this practice to certain settings will also be provided.

## 1. Introduction

Respiratory distress syndrome (RDS), formally known as hyaline membrane disease, is primarily an acute pulmonary process associated with immature lungs that are deficient in surfactant [1]. The absence or insufficient amount of surfactant causes increase surface tension in the alveoli leading to alveolar collapse and atelectasis. This leads to complications such as respiratory distress from increased work of breathing, hypoxia, ventilation-perfusion mismatch, and eventually respiratory failure. Aside from prematurity, risk factors for RDS include male sex, chorioamnionitis, a caesarean delivery without labor, and maternal diabetes [2]. RDS is inversely related to gestational age; occurring in about 100% of infants born <28 weeks’ gestation, about 30% of infants born between 28 and 34 weeks’ gestation, and about 1% and 14% of infants born after 34 weeks’ gestation [1,3,4]. Infants born at late-preterm (34 0/7 to 35 6/7 weeks’ gestation) are at higher risk for respiratory complications such as RDS than term infants. The Canadian Neonatal Network showed that in a cohort of 6636 infants who were admitted to the Neonatal Intensive Care Unit (NICU) between the gestational ages of 34 to 40 weeks, the incidence of RDS was as high as 14.2% in late-preterm (34 0/7 to 36 6/7 weeks’ gestation) infants, 7.2% in early term (37 0/7 to 38 6/7 weeks’ gestation) infants, and 4.5% in term (39 0/7 to 40 6/7 weeks’ gestation) infants [5]. 

Newborn infants presenting with persistent respiratory distress will require admission to a NICU or a special care newborn nursery. The respiratory symptoms of RDS can vary from mild to severe. Therefore, respiratory support can range from noninvasive support such as nasal cannula (NC) and nasal continuous positive airway pressure (CPAP) for mild to moderate RDS to invasive mechanical ventilation for severe RDS. One study that assessed term infants with RDS had 20% of infants on NC, 56% on nasal CPAP, and 25% on mechanical ventilation [6]. Additionally, admission to the NICU or special care nursery will cause separation of the mother-infant dyad and increase stress for the parents. The two most common diagnoses for respiratory distress are RDS and transient tachypnea of the newborn (TTN) [4,7]. However, the clinical findings of TTN and RDS in late-preterm and early term infants may be difficult to differentiate clinically. Both are associated with clinical signs and symptoms of respiratory distress such as grunting, tachypnea, retractions, nasal flaring, and possible oxygen requirements. One clinical difference is the time to resolution. TTN is a form of transitional delay, where fluid retention persists in the lungs and can resolve within 2 to 12 h. However, some cases of TTN can persist up to 72 h which is similar to the resolution of RDS [8]. Radiographically, TTN will have prominent perihilar vascular markings due to engorged periarterial lymphatics, edema of the interlobar septae, and fluid in the fissures [8]. Whereas in RDS, radiographic findings would be consistent with diffuse ground-glass opacities, air bronchograms, and decreased lung volumes. Aside from gestational age, TTN and RDS are more likely to occur in caesarean deliveries without labor due to decreased fluid absorption and for infants of diabetic mothers due to delay in fetal lung maturation and inhibition of surfactant expression secondary to fetal hyperinsulinism in response to elevated fetal glucose [9,10]. 

**Figure 1:**
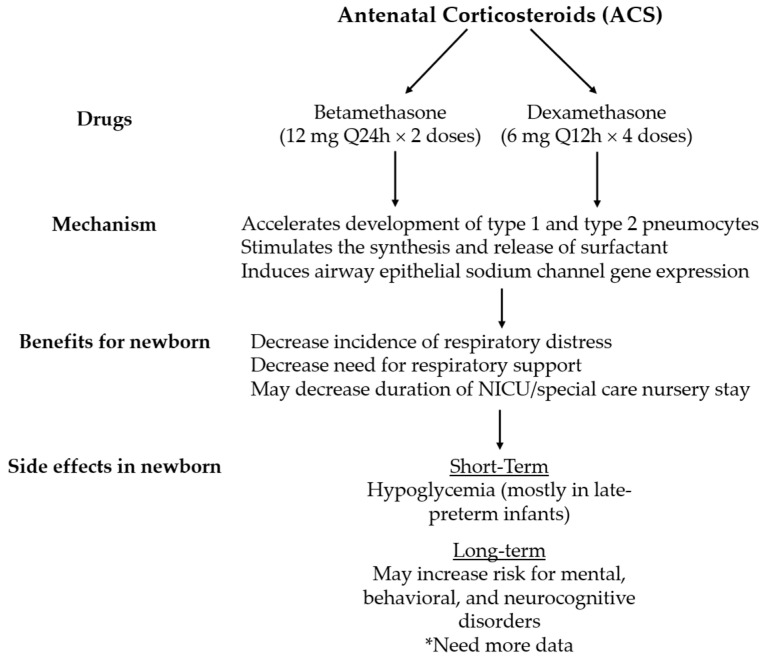
Outlines the corticosteroid agents, mechanism of corticosteroids in utero, and the benefits and side effects of ACS for the newborn infant. * Findings based on two retrospective studies; future research in long-term outcomes are needed.

Administration of maternal antenatal corticosteroids (ACS) is a well-adopted practice for preterm pregnancies <34 weeks’ gestation. Benefits for preterm infants include reducing the risk of perinatal death, neonatal death, RDS, and intraventricular hemorrhage (IVH) [11]. Specifically, for RDS, ACS facilitates surfactant production which can improve gas exchange and lung compliance. They also promote fluid absorption in the fetal lungs by upregulating gene expression for the epithelial Na+ channels (Figure 1) [12]. Both of these mechanisms can be beneficial to reduce the incidence of RDS and/or TTN in the late-preterm and early term infants [13]. The purpose of this review is to discuss the risk and benefits of the administration of ACS for late-preterm and early term pregnancies and to ensure that ACS is administered to the appropriate population setting. 

## 2. Antenatal Corticosteroid Agents and Current Recommendations

Two types of corticosteroids are used antenatally to facilitate lung maturation: betamethasone and dexamethasone. The cellular mechanisms of corticosteroids in facilitating lung maturation involve the induction of pulmonary beta-adrenergic receptors, acceleration of the development of type 1 and 2 pneumocytes, and upregulation of gene expression for the epithelial Na+ channel (Figure 1). This leads to increased surfactant production and excretion which allows for improvement in gas exchange and lung mechanics, and increased absorption of alveolar fluid prior to delivery [12,14,15]. The current World Health Organization (WHO) recommendation is to give intramuscular injections of either dexamethasone or betamethasone for a total of 24 mg in divided doses; e.g., 6 mg of dexamethasone every 12 h for a total of 4 doses or 12 mg of betamethasone every 24 h for a total of 2 doses [16]. While antenatal corticosteroid use has been endorsed most recently in 2016 by the American College of Obstetricians and Gynecologists (ACOG) and in 2017 by the American Academy of Pediatrics, the data do not support betamethasone use over dexamethasone [17,18]. Both corticosteroids have equal potency in maintaining glucocorticoid effects without mineralocorticoid activity, and each has weak immunosuppressive capabilities in the short term. The only difference is that betamethasone has a longer half-life because of its decreased clearance and larger volume of distribution [17,19,20]. Both betamethasone and dexamethasone remain the recommended therapies in ACOG’s committee opinion on corticosteroid administration for fetal maturation [17]. Each has shown long-term pulmonary and neurologic benefits when administration occurs for pregnancies less than 34 0/7 weeks of gestation [21,22]. In 2013, a Cochrane review of ten trials found no difference in perinatal death between these two antenatal corticosteroids; however, the intraventricular hemorrhage incidence was lower with dexamethasone administration [23]. One National Institute of Child Health and Human Development Neonatal Research Network (NICHD) study did find an increased likelihood of unimpaired neurologic state in extremely low birth weight infants at corrected ages of 18–22 months when betamethasone, versus dexamethasone, was administered antenatally [24]. As a result of the variance in reports, there are not enough data to recommend one of these corticosteroids over the other for this indication; therefore, either regimen is acceptable [17].

Both the ACOG and the Society of Maternal Fetal Medicine (SMFM) recommends considering the administration of ACS to expecting mothers between 34 0/7 weeks and 36 6/7 weeks’ gestation who are at high risk for preterm delivery within 7 days if they have not received a prior course of antenatal steroids [13,17]. High risk for preterm delivery is defined by preterm labor (with at least 3 cm of dilation or effacement of 75%) or preterm premature rupture of membranes (Table 1). Internationally, Australia and New Zealand’s clinical practice guideline recommends a single course of ACS in women only up to 34 6/7 weeks’ or less gestation if birth is expected within the next seven days [25]. This practice is similar to Canada’s practice guideline. However, Canada’s practice guideline also states that ACS administration can be considered for pregnancies between 35 0/6 to 36 6/7 weeks’ gestation in unique clinical situations after risks and benefits have been discussed with the mother [26]. The expansion of the ACS practice to high risk late-preterm delivery was positively impacted by the results of an NICHD study, Antenatal Betamethasone for Women at Risk for Late Preterm Delivery (ALPS) Trial [27].

## 3. Antenatal Corticosteroids for Late Preterm Delivery

Maternal factors associated with increased risk for preterm birth include prior preterm delivery, multiple gestations, diabetes or gestational diabetes, substance use, hypertension, and infection [28]. In 2016, the Antenatal Betamethasone for Women at Risk for Late Preterm Delivery (ALPS) Trial results were published. The study was performed due to the rising incidence of late-preterm deliveries [29]. It investigated the benefit of administering betamethasone in decreasing neonatal respiratory adverse outcomes and other complications [27]. The ALPS trial (*n* = 2831) was a multicenter, randomized trial (the largest to date) that included women with singleton pregnancies from 34 0/7 weeks to 36 5/7 weeks’ gestation who were at high risk for late-preterm delivery. The trial assessed a course of betamethasone (2 doses of 12 mg each, administered 24 h apart) versus placebo. The most common indication for enrollment was preterm labor (28%), followed by delivery for gestational hypertension or preeclampsia (26%) and ruptured membranes (22%) [27]. The primary composite outcome assessed the incidence of respiratory support required (defined as CPAP or high-flow NC for at least 2 h, supplemental oxygen with fraction inspired oxygen of at least 30% for at least 4 h, mechanical ventilation, or extracorporeal membrane oxygenation) in the first 72 h of life, stillbirth or neonatal death within 72 h of birth. Late-preterm infants born to pregnant women who received betamethasone versus placebo had a reduction in the primary outcome; 11.6% versus 14.4%, respectively (relative risk (RR), 0.80; 95% confidence interval (CI) 0.66 to 0.97; *p* = 0.02). There was a notable decrease in use of CPAP when betamethasone was administered compared to placebo; 10.2% vs. 13.1%, respectively; *p* = 0.01. This extends to CPAP use longer than 12 h; 6.5% vs. 12.1%; *p* < 0.001. Of note, other significant findings include: decrease in need for resuscitation at birth (14.5% vs. 18.7%; *p* = 0.003), surfactant use (1.8% vs. 3.1%; *p* = 0.03), and decrease in composite incidence of TTN, RDS, and apnea (13.9% vs. 17.8%; *p* = 0.004) [27]. Although the length of NICU stay was similar in both groups, the duration of stay ≥3 days was lower in the betamethasone group (32.9% vs. 37%; 0.89 (0.80–0.98; *p* = 0.03). There was no difference in maternal outcomes such as mode of delivery, clinical chorioamnionitis, or endometritis. One adverse effect for the newborn was a significantly higher rate of hypoglycemia (glucose < 40 mg/dL) in the betamethasone group compared to placebo (24.0% vs. 14.9%; RR 1.61, 95% CI 1.38–1.88, *p* < 0.001). However, this is a common complication in late-preterm infants and the infants who were hypoglycemic in this trial did not have a longer stay in the hospital [30]. Additionally, a study by Balci et al. in 2010 (*n* = 100) was a prospective trial where the treatment group received a 12 mg single dose of betamethasone. The rate of RDS was 4% in treatment group vs. 12% in the control group (OR 0.21; 95%CI 0.04–1.08; *P* = 0.046). This opens up a research interest of asking the question if a single dose is just as efficacious as a single course with two doses to reduce the incidence of RDS, or even perhaps TTN. Overall, current evidence suggest ACS is only recommended for women at high risk for late-preterm delivery using the indications from the ALPS trial. The ALPS trial did not perform a subgroup analysis of infants born to mothers with gestational diabetes or hypertension. Additionally, currently, there are no studies that have assessed late-preterm infant outcomes when antenatal steroids were given to mothers with gestational diabetes, hypertension, or pregnant with intrauterine growth restricted (IUGR) infants. Therefore, further evidence is needed before this can be extended to all late-preterm pregnancies. 

## 4. Antenatal Corticosteroids for Early Term Elective Caesarean Delivery

Infants born via elective caesarean delivery at 37 0/7 weeks to 38 6/7 weeks gestation are at increased risk for respiratory distress [31,32,33]. Labor induces catecholamines that help with the absorption of fetal lung fluid. However, with elective caesarean delivery, there is a lack of labor leading to retention of fluid in the newborn lungs which can result in respiratory distress. Although over the past decade the rate of caesarean delivery has been relatively stable, caesarean delivery still accounts for about 30% of deliveries [34]. One approach to preventing respiratory distress in the newborn infant is to reduce the incidence of caesarean delivery without labor. If caesarean delivery is indicated, ACOG recommends scheduling these deliveries at 39 weeks’ gestation or later or waiting for the onset of spontaneous labor. However, for those undergoing elective caesarean deliveries occurring between 37 and 38 6/7 weeks’ gestation, ACS might be beneficial in the short term for the newborn. 

An earlier study performed in 2005 by the Antenatal Steroids for Term Elective Caesarean Section (ASTECS) research team assessed whether antenatal steroids reduce respiratory distress in infants delivered by elective caesarean (*n* = 819) [35]. The treatment group received two intramuscular doses of 12 mg betamethasone 48 h prior to delivery, while the control group received treatment as usual (no antenatal steroids). The trial result showed that the incidence of NICU admission with respiratory distress was 2.4% in the treatment group vs. 5.1% in the control group (RR 0.46, 95% CI 0.23 to 0.93, *p* = 0.021); however, they were not able to demonstrate a difference in TTN or RDS. Another randomized trial by Ahmed et al. (*n* = 224) administered prophylactic antenatal steroids at 37 weeks gestation prior to term elective caesarean at 37 weeks or beyond [36]. In this study, neonates in the treatment group had a lower overall incidence of respiratory distress morbidity, 7.9% versus 23% when compared to the control group. The most significant benefit of steroid administration was noted in infants born between 37 0/7 and 37 6/7 weeks gestation. A randomized trial performed by Nada et al. (*n* = 1290) supports this finding in particular for infants born between 38 to 38 6/7 weeks gestation via elective caesarean. The treatment group who received three doses of antenatal steroids (dexamethasone 8 mg/dose every 12 h, 48 h before delivery) had a lower incidence of NICU admission for respiratory distress; 19/616 (3.1%) vs. 41/611 (6.7%) in the control group (RR 0.46, 95% CI 0.27–0.78, *p* = 0.003) [37]. However, the beneficial effect of antenatal steroids wanes past 39 0/7 weeks gestation. Hansen et al. suggest that there is decreasing risk of respiratory morbidity in the newborn as gestational age increases. The study suggests that a significant reduction in neonatal respiratory morbidity may be obtained if elective caesarean delivery is postponed to 39 weeks of gestation [33]. In general, delivery at 39 weeks’ gestation is most beneficial for the newborn. Although some data confer benefits for administering ACS in elective early term caesarean deliveries, this practice cannot be recommended and should not extend towards any early term deliveries. This is due to possible long-term adverse effects, which will be discussed in the next session. Therefore, further research is warranted to weigh the risk versus benefits before this practice can be adopted, even in the scheduled elective early term caesarean deliveries. 

## 5. Short- and Long-Term Effects

Antenatal corticosteroid is beneficial in the short-term (Figure 1). The decreased rate of respiratory distress will potentially reduce the use of CPAP and/or admission rates to the special care nursery or NICU. For singleton mothers who are at risk for late-preterm delivery, this can be beneficial for several reasons. First, a reduction in admission can decrease the separation time of the infant from the mother. Reducing mother-infant dyad separation leads to better bonding, breastmilk production, and feeding tolerance, in addition to possibly shortening hospital stay duration. Second, this can be economically favorable by optimizing healthcare costs. A cost-effective analysis was performed assessing total mean woman-infant–pair cost in those who received antenatal betamethasone versus placebo. The maternal costs included the cost of betamethasone treatment if given and outpatient visits or inpatient admissions. The newborn costs included NICU stay with or without the need for respiratory support (e.g., infants who did not have respiratory distress but had hypoglycemia which required NICU stay). This analysis found that treatment with betamethasone was associated with a total cost of $4681 which was significantly less than the mean cost of $5379 in the placebo group (difference of $698; 95% CI, $186–$1257; *p* = 0.02) [38]. Lastly, neonates who develop respiratory distress may need respiratory support such as CPAP. However, the use of CPAP can increase the risk of developing air-leak syndromes in infants. Rates of pneumothorax ranging from 9 to16% with the use of CPAP in neonates have been reported [39]. Therefore, infants born at hospitals that do not have the capability of providing long-term respiratory support such as CPAP will require transport to another facility which is an added healthcare cost and stress to the family. Perhaps the benefits of administering ACS can be best exhibited in resource-limited settings, therefore further cost analysis studies will help. Another significant short-term complication of ACS is hypoglycemia for late-preterm infants, which is already commonly seen in this population. Of note, the randomized trials which administered ACS to mothers undergoing elective caesarean did not assess the incidence of neonatal hypoglycemia in their studies. 

Although ACS has been shown to provide short term benefits, clinicians should be hesitant to universally adopt this practice due to unknown long-term effects of ACS, particularly regarding neurologic outcomes (Figure 1). A population-based retrospective cohort study performed in Finland studied if ACS treatment is associated with mental and behavioral disorders in children born at term (≥37 weeks’ gestation) and preterm (<37 weeks’ gestation) [40]. The median length for follow-up was 5.8 years. For infants born at term, there was a higher risk for mental and behavioral disorders in the treatment group compared to non-treatment; (8.89% vs. 6.31%; absolute difference, 2.58% [95% CI, 1.92–3.29%]; hazard ratio (HR) 1.47 [95% CI, 1.36–1.69]). Similar results were concluded in another population-based retrospective cohort study performed in Canada [41]. The primary outcome assessed in this study was proven or suspected neurocognitive disorder. At 5 years of age, the primary outcome was higher among infants exposed to ACS (*n* = 5423) compared with non-exposed infants (*n* = 523,782): 61.7% vs. 57.8%, respectively (*p* < 0.001; number needed to harm (NNH) = 25, 95% CI 19 to 38; adjusted HR 1.12, 95% CI 1.08 to 1.16). However, the long-term data from the ASTECS trial did not show any difference in neurological outcomes [42]. ASTECS’s follow-up study used strengths and difficulties questionnaire (SDQ) which measures hyperactivity, emotional symptoms, conduct problems, peer problems, and social behavior. From the betamethasone group, 12% were reported to have learning difficulties compared to 14% in the placebo group. All of these results should be interpreted with caution. For the retrospective studies from Finland and Ontario, the timing, type, and amount of ACS given were not known. The long-term follow-up in ASTECS was low at 46% which may have impacted the findings. Unfortunately, the potential for long-term effects given the aforementioned findings cannot be ignored. 

## 6. Discussion

Although the incidence of RDS decreases with gestational age, late-preterm and early term infants can still have respiratory distress consistent with RDS and TTN. When interventions are required to help infants with respiratory distress, it can add emotional stress to the parents and financial strains to both the family and healthcare system (especially in resource-limited areas unable to provide respiratory support to newborn infants). The benefits of prophylactic antenatal corticosteroids may extend towards late-preterm and early term infants. ACS given for high-risk preterm delivery has been shown to reduce the composite incidence of TTN, RDS, and apnea by the ALPS trial [27]. If delivery at 39 weeks’ gestation is not possible, for scheduled early term caesarean deliveries, multiple randomized control trials have shown the benefit of ACS reducing respiratory morbidities in the newborn infant [35,36,37].

As expected, there are practice variations throughout the world for ACS. This may be due to the question of whether the short-term benefits outweigh the long-term risks. As shown by the aforementioned retrospective studies, there is a potential risk for behavior disorders or neurocognitive disorder. Therefore, widespread adoption for ACS administration for all late-preterm and early term pregnancies cannot be recommended at this time. As stated by ACOG and SMFM, antenatal corticosteroids can be beneficial for pregnancies at high risk for late-preterm delivery. Similar to the Canadian practice guideline, we recommend having a discussion with the mother about the risk and benefits. For early term deliveries, we believe that caution is warranted as short-term benefits may not outweigh the long-term risks. Therefore, it is not recommended to give ACS to all early term pregnancies. Perhaps the approach should be encouraging elective caesarean deliveries to be scheduled at greater than 39 weeks’ gestation, if possible, as the incidence of respiratory morbidities are lower at term. The long-term outcomes from the ALPS trial will be beneficial to clinical practice overall. However, more research studies and data on long-term outcomes of ACS administration for early term deliveries are still needed. 

Other research gaps exist. For example, although completing a course of betamethasone or dexamethasone at a total dose of 24 mg is effective, could a lesser dose provide a similar benefit? To address this question, a randomized, multicenter trial (BETADOSE) assessing full versus half dose of antenatal betamethasone to prevent severe neonatal RDS associated with preterm birth is currently ongoing. Although the study population is targeted for <34 weeks gestation, if the data from this study proves to be safe and effective, this can lead to further studies for higher gestational ages [43]. Other research gaps include assessment of ACS use in multiple gestation pregnancies as well as additional studies looking at long-term outcomes. Additionally, IUGR infants are at increased risk for developing RDS, especially those born at greater than 29 weeks’ gestation [44]. Maternal factors associated with IUGR include uteroplacental insufficiency, chronic maternal disease (such as hypertension, lupus, connective tissue disorders, antiphospholipid syndrome), and preeclampsia. Therefore, a randomized control trial comparing antenatal corticosteroid administration versus placebo to mothers with IUGR infants in the late-preterm period could help expand the criteria for ACS use. Lastly, a cost-analysis of using ACS in low-resource settings may be beneficial. In these settings, the short-term effects of reducing respiratory distress in newborn infants may outweigh the long-term risks. Deliveries occurring in hospitals unable to provide adequate treatment for neonates with respiratory distress can lead to transfer to another facility or further complications if the respiratory support is inappropriately used. 

## 7. Conclusions

Prophylactic administration of antenatal steroids can help decrease respiratory morbidity in late-preterm and early term infants. This will help reduce infant/parent separation and possible complications that infants may experience when receiving respiratory support or during a hospital stay. However, this short-term benefit does not outweigh possible long-term adverse effects on neurological outcomes. Additional research studies and long-term outcome data are needed before routinely administering antenatal corticosteroids to all pregnancies in the late-preterm and early term periods. Unlike the practice for administering ACS in preterm infants less than 34 weeks’ gestation, ACS practice for greater than 34 weeks’ gestation should be limited to certain situations. The administration of antenatal corticosteroids in late-preterm infants should only be given to women who are at high risk for preterm delivery and expected late-preterm delivery for another indication that was studied in the ALPS trial (Table 1). Administration of ACS for elective scheduled early term caesarean deliveries should not be considered or recommended at this time. 

## Figures and Tables

**Table 1:** Clinical practice considerations for administration of antenatal corticosteroids for late-preterm and early term pregnancies.

Population	Antenatal steroid	Indication (s)	Research Gaps
Late-Preterm (34 0/7–36/7 weeks)	Betamethasone or dexamethasone (IM, 24 mg total)	- ONLY for high risk for preterm delivery which is defined as: ○ Preterm labor (with at least 3 cm of dilation or effacement of 75% ○ Preterm premature rupture of membranes - If neither of the above apply, high risk can be defined as expected preterm delivery for any other indications studied by the ALPS Trial [27] - Consider discussion with mother about risks versus benefits	- Multiple gestation - Pregestational diabetes - Long-term outcomes of the neonates - Low-resource settings - IUGR infants
Early Term (37 0/7–38 6/7 weeks)	- Not recommended at this time for any pregnancies or deliveries at this gestational age period. - Long-term adverse effects may outweigh short-term benefits.		

## Abbreviations

ACOG	American College of Obstetricians and Gynecologists
ALPS	Antenatal Betamethasone for Women at Risk for Late Preterm Delivery
ACS	Antenatal corticosteroids
ASTECS	Antenatal Steroids for Term Elective Caesarean Section
CI	Confidence interval
CPAP	Continuous positive airway pressure
HR	Hazard ratio
IUGR	Intrauterine growth restriction
IVH	Intraventricular hemorrhage
NC	Nasal cannula
NICU	Neonatal Intensive Care Unit
NNH	Number needed to harm
RR	Relative risk
RDS	Respiratory distress
SMFM	Society of Maternal Fetal Medicine
SDQ	Strengths and difficulties questionnaire
TTN	Transient tachypnea of the newborn
